# The Role of the IL-12 Cytokine Family in Directing T-Cell Responses in Oral Candidosis

**DOI:** 10.1155/2011/697340

**Published:** 2010-10-24

**Authors:** Xiao-Qing Wei, Helen Rogers, Michael A. O. Lewis, David W. Williams

**Affiliations:** Tissue Engineering and Reparative Dentistry, School of Dentistry, Cardiff University, Heath Park, Cardiff CF14 4XY, UK

## Abstract

*Candida albicans* is an opportunistic fungal pathogen that normally exists as a harmless commensal in humans. In instances where host debilitation occurs, *Candida* can cause a range of clinical infections, and whilst these are primarily superficial, effecting mucosal membranes, systemic infections can develop in severely immunocompromised individuals. The mechanism of host immunity during commensal carriage of *C. albicans* has been intensively studied. In this paper, we present the most recent information concerning host recognition of *C. albicans* leading to cytokine production and the subsequent T-cell responses generated in response to *C. albicans*. Particular focus is given to the role of the IL-12 cytokine family including IL-12, IL-23, IL-27, and IL-35, in host immunity to *Candida*. CD4^+^ T-cells are considered crucial in the regulation of immunity and inflammation. In this regard, the role of Th1/2, helper cells, together with the recently identified Th17 and Treg cells in candidosis will be discussed. Understanding the detailed mechanisms that underlie host immunity to *Candida* not only will be of benefit in terms of the infections caused by this organism but could also be exploited in the development of therapeutic interventions for other diseases.

## 1. Introduction

*Candida albicans* is normally a harmless commensal fungus of humans and has been shown to colonise up to 70% of individuals without any detriment to health [1, 2]. During commensal carriage, a balance exists between the body's own defence systems and the continued persistence of the organism at a level that does not induce disease. However, in instances where there is disruption to this equilibrium, either through environmental factors that promote the growth of *Candida* or through a weakening of the host's immune system, then proliferation of *Candida* and subsequent infection can arise. Clear examples of this occur in HIV-infected individuals or those patients receiving chemotherapy for treatment of cancer [3–5]. In these situations, a depletion of host T-cell function and that of other cells involved in establishing an appropriate immune response occurs with the result that candidosis can ensue. 

Candidosis most frequently presents as superficial lesions of moist mucosal membranes, particularly those of the vagina and oral cavity. However, in severely immunocompromised individuals and particularly where there is neutropenia, systemic infections can develop which are often fatal. Most immunocompetent individuals have an underlying acquired immunity which is thought to prevent dissemination of mucosal candidal colonisation through the body. Individuals who are existing carriers of *Candida* are however deemed to be at an increased risk of developing systemic forms of the disease. The type of immune response generated is key in determining whether *C. albicans* clearance, infection, or commensal carriage occurs. 

Oral candidosis is not a single disease entity and four distinct clinical forms of primary infection are recognised. Pseudomembranous candidiasis frequently occurs in newborn babies and in these instances is associated with an immature immune system. In older individuals the condition occurs when there is mild debilitation or local immunosuppression in the host as seen in asthma sufferers who use a corticosteroid inhaler as part of their treatment regime [6]. With the advent and escalation of HIV infection and AIDS, chronic forms of pseudomembranous candidosis are evident, which frequently reoccur despite administration of antifungals. 

Acute erythematous candidosis is an infection that presents as a painful reddened lesion on the dorsum of the tongue and is associated with an overgrowth of *Candida* following broad spectrum antibiotics. Such lesions tend to resolve spontaneously after antibiotic therapy has been completed [7]. Chronic erythematous candidosis is the most prevalent form of oral candidosis affecting over 65% of denture wearers, often asymptomatically. It is thought that poor denture hygiene contributes to the infection that occurs on the palate beneath the upper fitting surface of the denture. Chronic hyperplastic candidosis is another chronic infection that presents as a white lesion on the oral mucosa, most frequently bilaterally on the buccal mucosa. The infection is particularly significant as it is associated with malignant change at the lesional site, although the role of *Candida* in this process remains unclear [8].

In addition to these primary infections, secondary oral infections are recognised and include angular cheilitis and median rhomboid glossitis. Angular cheilitis presents as lesions in the angles of the mouth from which *Candida albicans* is frequently recovered, often in combination with the bacterium *Staphylococcus aureus *[9]. Median rhomboid glossitis presents as a chronic tongue lesion within which *Candida* is detected by biopsy in over 85% of cases. 

Chronic mucocutaneous candidiasis (CMC) is a collective term for a range of syndromes that result in the persistent occurrence of severe and diffuse cutaneous candidal infections. These infections manifest on the skin, nails, and mucous membranes (including the oral cavity) of CMC patients. Invariably, there is an underlying dysfunction in cell-mediated immunity that leads to the occurrence of CMC [10].

Neutrophils and macrophages are major components of the innate immune response and play an important role in the control of mucosal *C. albicans* colonisation. These cells are responsible for the phagocytosis of *Candida* and can kill the fungus through release of reactive oxygen species (ROS). Cytokines produced by T-cells, such as IFN-*γ* and IL-17 will promote neutrophil and macrophage activity against *Candida* [11, 12]. Neutrophils and macrophages recognise *C. albicans* via pattern recognition receptors (PRRs) and following recognition will also produce an array of cytokines and chemokines.

Dendritic cells (DCs) function as professional antigen presenting cells (APCs) that detect the presence of *Candida* at mucosal sites. DCs will interact with the fungus and become activated, with phagocytosis being the first action of the activated cell. After phagocytosis, DCs migrate to the nearest lymph node where the *Candida* antigen is processed and presented by the DCs to naïve CD4^+^ T-cells [13–18]. The naïve T-cells then differentiate into mature effective T-cells under the direction of the DCs [19]. Examples of such differentiated T-cells include T-helper 1 (Th1), T-helper 2 (Th2), T-helper 17 (Th17), and regulatory T-cells (Tregs). The latter serve to control T-cell responses to avoid an overreactive immune response [20], and in this manner, an inducible Treg cell development can balance pathogenic and physiologic host immune responses [21–24]. 

Activated DCs produce a variety of cytokines to direct T-cell differentiation, and the IL-12 family of cytokines (IL-12, IL-23, IL-27, and IL-35) would appear to play an important role in this T-cell differentiation [25–27]. It is the relative level and type of cytokines that are expressed by the DCs into the local environment that will ultimately determine type of T-cell response (Th1, Th2, and Th17) generated. In this paper, we will discuss the role that members of the IL-12 cytokine family play in the host immune responses and their possible relationship in effecting either tolerance of fungal colonisation or the development of oral infection.

## 2. *Candida albicans* Recognition by Host DCs

As previously mentioned, the main function of DCs is to act as professional APCs that link the innate and adaptive host immune responses in combating host invading pathogens. Human peripheral blood-derived DCs are the main source of mucosal DCs, and it is these immature cells that will initially recognise *Candida* through various surface protein antigens and mannose and *β*-glucans in the fungal cell wall [5].

The initial antigen recognition by DCs is mediated through the expression of pattern-recognition receptors (PRRs) on the surface of DCs. The nature of this interaction between DCs and the fungus is the key factor in determining whether immune activation or tolerance occurs [8]. The first group of PRRs identified were the toll-like receptors (TLRs) and to date, 10 of these TLRs have been identified in humans, with TLR2, TLR4 TLR6, and TLR9 implicated in host cell recognition of *C. albicans *[28–30]. TLRs are type I membrane proteins and are either homodimer or heterodimer receptors, recognising various pathogen antigens including proteins, cell wall sugars, or DNA/RNA molecules (collectively referred to as pathogen-associated molecular patterns or PAMPs). Recently a new group of type II cell membrane molecules have been linked to the recognition of fungi by host cells, and these are referred to as C-type lectin-like receptors (CTLLRs) [31]. Dectin-1 and 2 are examples of CTLLRs and interact, respectively, with the *β*-glucan and mannose components exposed in the *C. albicans* cell wall [32, 33]. Binding of dectin-1 to *β*-glucan components of *C. albicans* will trigger host immune cell responses, and signalling is thought to be mediated through the Syk kinase pathway and the transmembrane adaptor caspase recruitment domain 9 (CARD9) leading to induction of the cytokines IL-23 and IL-6, but not IL-12 [34]. Interestingly in CMC patients, a CARD9 point mutation that leads to the generation of a premature termination codon (Q295X) has recently been identified [35]. 

Synergistic effects of dectin-1 with TLR2 and TLR4 signalling have been shown to enhance proinflammatory cytokine production by human peripheral blood monocytes (PBMCs) [36]. Recently, mutation in the human dectin-1 gene was identified in 4 women within a single family, who were all suffering from vulvovaginal candidosis. Monocytes isolated from these homozygote mutant patients all exhibited significantly reduced binding to *C. albicans*. These cells also produced less IL-6 and IL-17 cytokines [37].

## 3. The Role of the IL-12 Cytokine Family in Host Immunity

**Figure 1:**
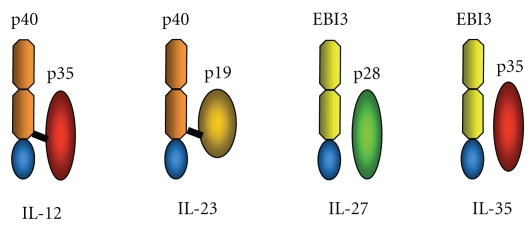
Schematic representation of structure of members of the IL-12 famil.

Many cytokines can be produced by APCs, although it is those belonging to the IL-12 family that would appear to be significant in controlling T-cell differentiation. This family of cytokines consists of at least 4 heterodimeric protein members and includes the cytokines IL-12, IL-23, IL-27 and IL-35 (Figure 1). 

The IL-12 cytokine is composed of two protein subunits called p35 and p40 and was the first cytokine shown to be able to drive the differentiation of naïve T-cells into Th1 cells [38, 39]. The IL-23 cytokine also shares the same p40 subunit as IL-12, but in IL-23 this is combined with a p19 subunit. IL-23 was discovered almost 10 years after IL-12 [40] and has also been implicated in the later stages of Th1 cell differentiation. Followup studies have subsequently shown that IL-23 is in fact, functionally distinct from IL-12, as it not only acts on Th1 cells, but also stimulates Th17 cells to produce IL-17 [30, 41]. Interestingly, IL-23 also acts on macrophages to drive osteoclast development in inflammation-mediated bone pathology [42]. 

The IL-27 cytokine comprises of a p40-related protein referred to as EBi3 (Epstein-Barr virus induced gene 3) together with a subunit known as p28, (a p35-related protein). The EBi3 gene was first identified in 1996, following its expression during B-lymphocytes infection with the Epstein Barr Virus. The gene product is a secreted glycoprotein related to p40 [43]. Later, during research investigating IL-6 homologous proteins, its partner subunit, p28 was discovered. The p28, protein was only found to be efficiently secreted when coupled with EBI3. Following this discovery, the heterodimeric protein of EBi3 and p28 was named IL-27 [44]. 

Initially, the function of IL-27 was again thought to be similar to IL-12 in driving early stage Th1 cell development. Further study has however shown that IL-27 is also able to induce IL-10 production from Treg cells which can inhibit Th17 responses [45, 46]. In this regard, IL-27 can suppress inflammation in inflammatory mouse disease models including the collagen induced arthritis (CIA) mouse model [46, 47]. 

In addition to associating with p28 in IL-27, the EBi3 subunit can also couple with p35 to facilitate secretion of the latter [48]. The function of the EBi3/p35 heterodimer was not known until relatively recently when studies using a recombinant EBi3/p35 protein in a rheumatoid arthritis mouse model [46] showed that joint inflammation was effectively resolved by the recombinant protein. It was suggested that the therapeutic mechanism of this recombinant protein was through induction of Treg cell development and IL-10 expression with the subsequent suppression of Th1 and Th17 responses [46]. Furthermore, in the absence of EBi3 or p35 subunit production, Treg cells are unable to resolve gut inflammation in mouse inflammatory bowel disease (IBD) [49]. EBi3 and p35 are both highly expressed by mouse Treg cells, thus seemingly providing a key component of the immune regulating function of these cells. In cases where Treg cells are deficient in either the EBi3 or p35 genes, a reduced ability to suppress effector T-cell proliferation is evident [49]. 

The EBi3/p35 heterodimeric protein has since been named IL-35 [49], and whilst the functional effect of IL-35 in mouse models has clearly been seen, the role of this particular cytokine in immune regulation of humans still remains unclear.

### 3.1. The Role of IL-12 in Candidosis

A critical role of IL-12 in the immune response during human fungal infection has been reported. The *β*1 subunit of the IL-12 receptor is utilised by both IL-12 and IL-23 for cell signalling transduction and is encoded by the IL12RB1 gene. Recently, two unrelated Mexican patients were found to share a common mutation in the IL12RB1, gene and both were found to suffer from uncontrolled Bacille Calmette-Guérin (BCG) and *C. albicans* infection resulting in their early deaths by the ages of 4 and 16 years [50]. 

DC maturation is essential in the generation of an immune response following *C. albicans* stimulation. Human PBMC populations contain a large number of immature DCs and when stimulated with *C. albicans*, IL-12p40 mRNA expression is not detected [51]. However, *in vitro* derived and matured DCs from PBMC do respond to *C. albicans* stimulation with subsequent IL-12 production, which in turn stimulates Th1 cell differentiation [52]. 

The importance of IL-12p40 in controlling fungal infection has been shown in *C. albicans* infected mouse models. Mannoprotein from the fungal pathogen *Cryptococcus neoformans* appears to protect mice from subsequent intravenous challenge with *C. albicans*. However neutralisation of the IL-12 cytokine using a rat anti-IL-12 antibody removes this protective effect, indicating an involvement of IL-12 in the process [53]. An IL-12 recombinant protein fused with *C. albicans* antigen (enolase) has also been reported as being able to protect mice during *C. albicans* infection [54]. 

Both mucosal and systemic models of mouse candidosis have been developed [55–57]. Based on these, it has been shown that IL-12p40 deficient mice exhibit significantly higher levels of *C. albicans* colonisation than their wild-type littermates. However, systemic infection with intravenously inoculation of *C albicans* appears unaffected by IL-12p40 deficiency [58].

### 3.2. The Role of IL-23 in Candidosis

Conceivably, the results obtained with the IL-12p40 gene knockout mice described above may also be the result of deficiencies in IL-23 production, since IL-12 and IL-23 share a protein subunit (IL-12p40). Recently, studies have shown that IL-23 may indeed play a key role in controlling vaginal candidosis, with the IL-23 cytokine, together with IL-6 readily detected in vaginal secretions of infected women [12, 59]. Even though DCs isolated from patients with candidosis demonstrate reduced maturation, they do have the ability to produce IL-23 and IL-6 [19], which seemingly can play a direct role in the maintenance of Th17 responses [60–62]. 

*Candida* infection in both immune competent and immunosuppressed mice generally leads to enhanced IL-23p19 expression. Immune competent mice produce a much higher and more prolonged expression of IL-23p19 mRNA at mucosal tissue sites compared with those mice that are immunosuppressed [12]. Mice that are Th17 and IL-17 receptor deficient develop an inappropriate immune response [63], and other results indicate that over production of IL-23 and IL-17 can impair the host immune system in its ability to combat *C. albicans infection*, despite mice exhibiting inflammation [41].

### 3.3. Involvement of IL-27 and IL-35 in Candidosis

IL-27 and IL-35 are cytokines that regulate the production of IL-17 and IL-10 from Th17 and Treg cells, respectively. IL-27 and IL-35 both suppress IL-17 production by Th17 cells and promote IL-10 production by Treg or T suppressor cells [45, 46]. Although IL-27 can enhance APC and DC maturation by stimulation of MHC I and II expression in human THP-1 monocytic cells [64], an autocrine-mediated regulation has, as yet, not been demonstrated. 

**Figure 2:**
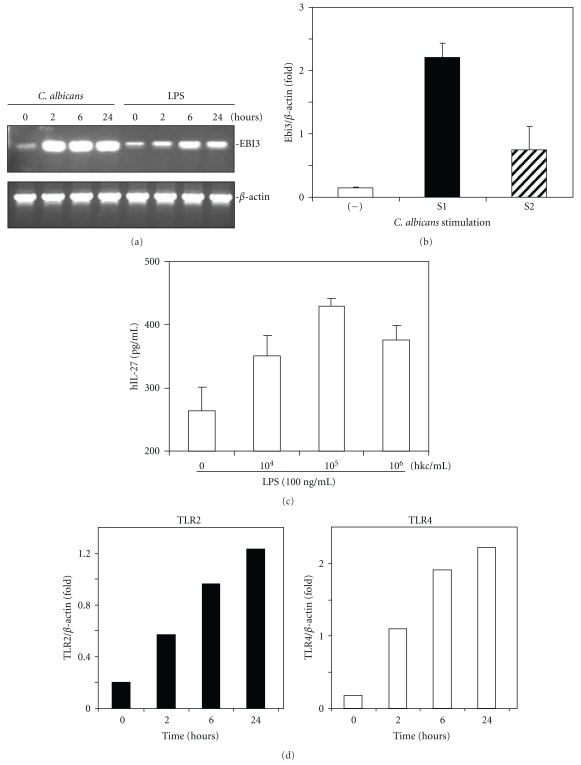
Heat-killed *Candida albicans *(HKC) stimulated THP-1 cell for Ebi3 and TLR2/4 expression. (a) THP-1 cells were stimulated with 1 × 10^5^ HKC /mL and 100 ng/mL LPS for indicated time. The Ebi3 mRNA was detected by RT-PCR. The RNA equal loading was shown by *β*-Actin RT-PCR. (b) THP-1 cells were stimulated with two clinical strains of *C. albicans* (S1 and S2) for overnight. The Ebi3 expression was presented by band density ratios of Ebi3 over *β*-Actin. (c) IL-27 cytokineS were measured by a human IL-27 ELISA (R&D system) in a cell culture with pretreated THP-1 cell with indicated increase density of heat killed Candida followed by low dose LPS stimulation. (d) TLR2 and 4 mRNA expression was increased by 1 × 10^6^ HKC /mL.

IL-12p35 knockout mice tend to have lower fungal burden and show no overt disease during oral *C. albicans* infection [63]. Since IL-12p35 is a component of IL-35, the result of this study demonstrated that the immunosuppressive effect of IL-35 can be countered by gene disruption, resulting in enhanced immunity against fungi infection. To date, there has been limited laboratory research into the role that IL-27 and IL-35 may have in the immune response to candidosis. Recent experiments performed in our laboratory (unpublished data) have shown that when THP-1 cells are challenged with heat killed *C. albicans,* an enhanced expression of EBi3 (a subunit of both IL-27 and IL-35) occurs compared with exposure to bacterial lipopolysaccharide alone (Figure 2(a)). Furthermore we have found that clinical strains of *C. albicans* exhibit strain variation in their ability to induce EBi3 mRNA expression in the THP-1 cells (Figure 2(b)). Prechallenging of THP-1 cell with heat killed *C. albicans* also leads to a detectable level of IL-27 expression after initial low dose LPS stimulation (Figure 2(c)), which can be explained by enhanced expression of TLR2 and TLR4 (Figure 2(d)).

## 4. T-cell Immunity in Controlling Fungal Infection

As previously mentioned, the binding of PRRs to PAMPs on the *Candida* cell surface activates DCs and other professional APCs. Activated APCs can phagocytose *C. albicans* and present its antigens with MHC II to T-cell receptors (TCRs) of naïve CD4^+^ T-cells. The interaction of the DC and T-cell usually occurs within lymphoid tissue immediately after migration of the APC from the site of infection. APC binding with naïve CD4^+^ T-cells will result in the directed differentiation and proliferation of various T-cell subsets. The type of subset of T-helper cells that is developed is dependent on the cytokine environment supplied by the APC which will have a major bearing on the ensuing immune response. 

Three signals are provided by the APC to the naïve CD4^+^ T-cell. The first is through interaction of the presented antigen and the TCR. The second is provided by costimulatory molecules on the APCs which bind to costimulatory receptors on T-cells. The third signal involves the production of cytokines by the APCs, which activate appropriate receptors on T-cell surfaces and induce T-cell differentiation [65–68]. Many cytokines can be produced by activated APCs, and these can either be pro- or anti-inflammatory [33, 34, 69]. Results from animal models clearly show that T-cells are pivotal in the control of *Candida* colonisation of the host [70–72]. 

Protective adaptive immune responses to *Candida* were initially thought to be mediated by CD4^+^ Th1 cells [15, 72], with Th2 cells associated with a failure to protect resulting in *Candida* infection [4, 73]. More recently, Th17-mediated immunity has been highlighted as a protective mechanism against *Candida* infection [74, 75]. 

### 4.1. Th1 and Th2 Cell Responses in Candidosis

By definition, a Th1 cell is a CD4^+^ T-cell that has the ability to respond to a specific antigen and produce the cytokines IFN*γ*, IL-2, and TNF*α*. Similarly, Th2 cells are CD4^+^T-cells that produce the cytokines IL-4, IL-5, IL-13, and IL-25 in response to antigen stimulation. It has been demonstrated that Th1 cells play an important role in the defence of mice against *C. albicans* infection [76, 77]. Studies that have neutralised IL-4 (a Th2 cytokine) production in mice resulted in an induced Th1 response with an associated resistance to *C. albicans* infection [78]. In addition, IFN*γ* receptor-deficient mice exhibit a greater susceptibility to *C. albicans* systemic infection. In cases where human peripheral blood derived DCs are stimulated with *C albicans* and cocultured with autologous CD4^+^ T-cells, a Th1 response is also elicited [52]. Defects in these Th1 cell responses lead to a significant impairment in host defences against *Candida* [79]. 

Although a protective role of Th1 responses has convincingly been showed in both humans and mice, contrasting results concerning Th1 or Th2 cytokine production at mucosal infection sites have been reported [71], and in some patients with candidosis, these cytokines may not even be detectable [80]. These findings may however be a result of different assay sensitivities in respective laboratories.

### 4.2. Th17 in Candidosis

Th17 cells produce the cytokines IL-17A, IL-17F, IL-21, and IL-22 [81]. Evidence for the importance of Th17 cells in protecting against *Candida* infection has been through experiments involving mice deficient in production of Th17 cells, the expression of IL-17, or for the receptor for IL-17. In such studies, the deficient mice exhibit greater susceptibility to oral infection with *C. albicans* [63]. Furthermore, human PBMCs from patients with chronic mucosal candidosis have been shown to exhibit reduced Th17 cell responses compared with PBMCs from healthy individuals after challenge with *C. albicans *[82, 83]. 

Mouse dectin-1 activated DCs direct Th17 cell development *in vitro*, and in a mouse candidosis model, CARD9-dependent Th17 development has been demonstrated [34]. IL-22 in mice plays a protective role against *C. albicans* infection when Th1 responses are impaired [84]. In addition, *C. albicans*-specific Th17 cells are readily detected in healthy individuals with a *C. albicans* mediated induction of Th17 responses observed via DCs [12]. Human monocyte derived DCs stimulated with the generally nonpathogenic *Saccharomyces cerevisiae* yeast induce cell-mediated Th1 responses, while *C. albicans* yeast or hyphae pulsed DCs shifted T-cell responses towards Th17 [85]. 

Hyphae of *C. albicans* have been shown to stimulate human monocyte-derived DCs to produce IL-23 and not IL-12. This is in contrast with *C. albicans* yeast which stimulate IL-12 production, with IL-23 only generated in response to high yeast concentrations. Such findings would suggest that it is the hyphal form of *C. albicans* that is responsible for triggering Th17 responses *in vivo*. Interestingly, human memory cells specific for *C. albicans* are derived from Th17 subsets that express the chemokine receptors CCR6 and CCR4 for skin and mucosal homing [86]. The exact function of this chemokine receptor expression in the control of *in vivo* Th17 cell migration does however remain unclear. Moreover, PBMCs from a CMC patient were found to produce lower levels of IL-17A and IL-22 in response to *C. albicans* stimulation compared with cells from a healthy individual. A decreased number of CCR6^+^ CD4^+^ T-cells have also been found in CMC patients [87].

### 4.3. Regulatory T-cells in Candidosis

Regulatory T-cells (Treg) are CD4^+^CD25^high^ T-cells which can suppress CD4^+^T effective cells, including the proliferation and differentiation of Th1 and Th17 cells. Such a suppression leads to reduced inflammatory responses against *Candida* in mucosal and disseminated infections [19, 28]. 

*Candida albicans* might be able to induce immune tolerance through interaction with DCs via Toll/IL-1 receptor domain-containing adaptor inducing IFN-*β* (TRIF) and STAT3-dependent signal transduction that activates Treg differentiation. Treg cells express TLR2 and proliferate under stimulation with TLR2 ligands and TCR activation. Moreover TLR2^−/−^ mice are more resistant to *C. albicans* infection and demonstrate significantly reduced IL-10 production. This would suggest that Treg cell function may be regulated directly by *C. albicans* to avoid unnecessary inflammatory responses [28, 29]. The balance between Th17 and Treg cells in mucosal tissue has been suggested as the determining factor for either commensal carriage or infection with *C. albicans *[13, 88]. However, human and animal models have yet to show any direct evidence that an over production of Treg cells and enhanced Treg function leads to *C. albicans* infection. Indirectly, Treg involvement in candidosis has been demonstrated using TNF*α* receptor-related gene (GITR) knockout mice. The wild-type mouse is associated with high expression of CD4^+^CD25^+^ T-cells (Treg). The GITR knockout mice have enhanced resistance to systemic *C. albicans* infection with an accompanying highly developed Th1 cell phenotype. Furthermore DCs produce elevated IL-12 levels when added to cultures of CD4^+^CD25^+^ T-cells from GITR knockout mice, when compared with wild-type mice [89]. This finding indicates that Treg cells may serve to suppress the protective Th1 role in candidosis. As previously mentioned, the cytokine IL-35 consists of the subunits EBi3 and IL-12p35 and would appear to promote Treg differentiation [46] and therefore control Treg immune suppression [49]. Mice deficient in the IL-12p35 gene are highly resistant to oropharyngeal candidosis [63], which again provides indirect evidence for the role of Treg in suppressing T-cell defensive responses against *C. albicans* infection.

**Figure 3:**
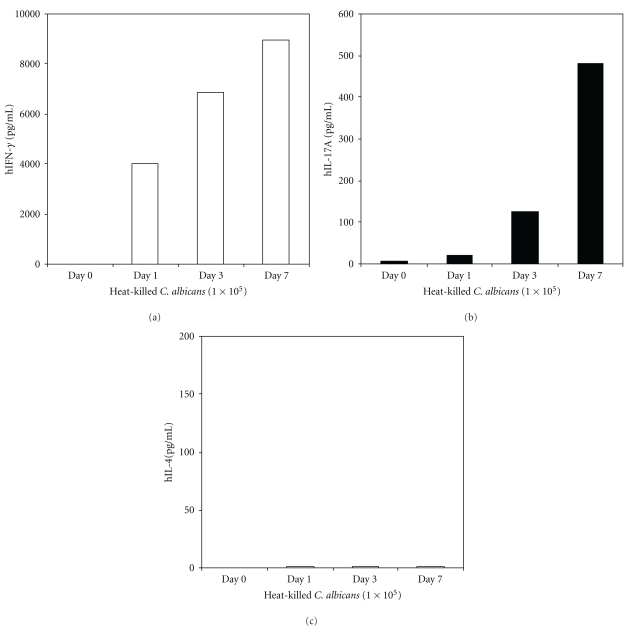
Peripheral blood monocytes from a healthy individual responded to C. albicans stimulation for Th1 cytokine (IFN*γ*) production, Th17 cytokine (IL-17) production, but not Th2 cytokine (IL-4) production. The cytokines were measured with CBA kit (BD bioscience).

T-cell responses can be generated by coculture of a pathogen's antigen with peripheral blood monocytes. After several days of culture, the antigen-specific T-cells can be recalled to produce cytokines, such as the Th1 cell cytokine IFN*γ* [90]. We have isolated human PBMC from a healthy donor and recalled the T-cell response using heat killed *C. albicans*. Predominant Th1 and Th17 responses based on the cytokine profiles were seen at days 1, 3, and 7 after culturing PBMC with heat killed *C. albicans* (Figure 3).

## 5. Summary

DCs are the key immune surveillance cells at mucosal sites and mediate their surveillance through cell surface expression of pattern recognition receptors (PRRs). The colonisation of mucosal surfaces by *C. albicans* can be detected through interaction of PRRs and surface antigens on *Candida*, which will ultimately lead to cell signalling and phagocytosis. Activated DCs migrate to local lymph nodes where the processed *Candida* antigens are presented to naïve CD4^+^ T-cells. T-helper and Treg cells will differentiate under the direction of cytokines produced by the DCs. The IL-12 cytokine family are largely involved in driving this T-helper cell development which in turn is essential for subsequent macrophage and neutrophil killing of *C. albicans*. Both Th1 and Th17 responses are considered protective, whilst Th2 and Treg responses may have a suppressive effect on host immunity against candidosis. The cytokine IL-12 drives Th1 development, whilst IL-23 is involved in directing and maintaining Th17 function. IL-27 has two effects in terms of regulating CD4^+^T-cell responses. On one hand, IL-27 assists IL-12 in the early stage development of Th1 cells; however, like IL-35, IL-27 also serves to suppress Th17 activity. The role of IL-12 cytokines in immune function against *Candida* is still not completely understood, and our current knowledge particularly for the novel IL-12 family members, IL-27 and IL-35, needs to be enhanced. Additional research is warranted in order to understand the molecular mechanism of host against *C. albicans* infection, which will not only benefit in the treatment of candidosis but could also offer therapeutic options in the treatment of other inflammatory conditions.
